# *In vitro *and *in vivo *MMP gene expression localisation by *In Situ*-RT-PCR in cell culture and paraffin embedded human breast cancer cell line xenografts

**DOI:** 10.1186/1471-2407-6-18

**Published:** 2006-01-24

**Authors:** Larisa M Haupt, Erik W Thompson, Ann EO Trezise, Rachel E Irving, Michael G Irving, Lyn R Griffiths

**Affiliations:** 1 Genomics Research Centre, Griffith University Gold Coast, School of Medical Science, Griffith University, Queensland, 4217,Australia; 2 IMCB, Biopolis, Singapore; 3 VBCRC Invasion and Metastasis Unit, St. Vincent's Institute of Medical Research and University of Melbourne, Department of Surgery, Melbourne, Victoria, 3065, Australia; 4 School of Biomedical Sciences, University of Queensland, St Lucia, Queensland 4072, Australia; 5 Institute of Health Sciences, Bond University, Queensland, 4229, Australia

## Abstract

**Background:**

Members of the matrix metalloproteinase (MMP) family of proteases are required for the degradation of the basement membrane and extracellular matrix in both normal and pathological conditions. *In vitro*, MT1-MMP (MMP-14, membrane type-1-MMP) expression is higher in more invasive human breast cancer (HBC) cell lines, whilst *in vivo *its expression has been associated with the stroma surrounding breast tumours. MMP-1 (interstitial collagenase) has been associated with MDA-MB-231 invasion *in vitro*, while MMP-3 (stromelysin-1) has been localised around invasive cells of breast tumours *in vivo*. As MMPs are not stored intracellularly, the ability to localise their expression to their cells of origin is difficult.

**Methods:**

We utilised the unique *in situ*-reverse transcription-polymerase chain reaction (*IS*-RT-PCR) methodology to localise the *in vitro *and *in vivo *gene expression of MT1-MMP, MMP-1 and MMP-3 in human breast cancer. *In vitro*, MMP induction was examined in the MDA-MB-231 and MCF-7 HBC cell lines following exposure to Concanavalin A (Con A). *In vivo*, we examined their expression in archival paraffin embedded xenografts derived from a range of HBC cell lines of varied invasive and metastatic potential. Mouse xenografts are heterogenous, containing neoplastic human parenchyma with mouse stroma and vasculature and provide a reproducible *in vivo *model system correlated to the human disease state.

**Results:**

*In vitro*, exposure to Con A increased MT1-MMP gene expression in MDA-MB-231 cells and decreased MT1-MMP gene expression in MCF-7 cells. MMP-1 and MMP-3 gene expression remained unchanged in both cell lines. *In vivo*, stromal cells recruited into each xenograft demonstrated differences in localised levels of MMP gene expression. Specifically, MDA-MB-231, MDA-MB-435 and Hs578T HBC cell lines are able to influence MMP gene expression in the surrounding stroma.

**Conclusion:**

We have demonstrated the applicability and sensitivity of *IS*-RT-PCR for the examination of MMP gene expression both *in vitro *and *in vivo*. Induction of MMP gene expression in both the epithelial tumour cells and surrounding stromal cells is associated with increased metastatic potential. Our data demonstrate the contribution of the stroma to epithelial MMP gene expression, and highlight the complexity of the role of MMPs in the stromal-epithelial interactions within breast carcinoma.

## Background

Human breast carcinoma (HBC) is the most predominant cancer in females worldwide. Breast cancers can be classified histologically based upon the types and patterns of cells of which they are composed. Carcinomas can be invasive, extending into the surrounding stroma or non-invasive, confined to the epithelial cells of ducts or lobules [1]. In conjunction with other protease systems such as the serine, cysteine and aspartyl proteases, members of the matrix metalloprotease (MMP) family of proteolytic enzymes degrade constituents of the extracellular matrix surrounding invasive breast carcinomas [2]. Currently, 28 human MMPs have been identified and classified according to both their substrate specificities and structural similarities. There are four major subgroups: i) interstitial collagenases; ii) gelatinases; iii) stromelysins; and iv) the membrane-type (MT) -MMPs [3,4]. Collectively, MMPs degrade all extracellular matrix proteins as well as a growing number of key regulatory genes such as cytokines, growth factors, cell surface receptors and adhesion molecules [5,6]. Although MMPs are expressed by tissues at various stages of development, they are typically absent in normal cells of the adult organism [2], and the high frequency at which MMP transcripts or proteins are detected in invasive tumours has implicated these enzymes in the establishment, growth, invasion and/or metastasis of tumours [6]. The expression of MMPs is usually tightly regulated, and a number of studies provide evidence of carefully controlled MMP involvement in developmentally regulated processes such as ovulation, embryogenic growth and differentiation, and organ development [6-8]. In general terms, MMP activity is regulated at least at three levels: transcription/translation, proteolytic activation of the zymogen, and inhibition of the active enzyme [2] with upregulation of each of these associated with pathological events [6]. Importantly in all mammalian cells except polymorphonuclear (PMN) leucocytes, most MMPs are not stored intracellularly but are rapidly secreted after biosynthesis and post-translational processing [9]. As a result it is difficult to identify their cellular origin using standard immunohistochemical techniques [10].

MT1-MMP is one of the membrane bound MMPs. *In vivo*, MT1-MMP expression has been localised to the stroma surrounding breast tumours [11,12], whilst *in vitro*, our recent data confirms previous studies where basal levels of MT1-MMP have been shown to be higher in the more invasive MDA-MB-231 cells as compared to the less invasive MCF-7 cells [13,14]. MT1-MMP has been shown to activate pro-gelatinase-A (proMMP-2) in human breast carcinoma cells [15] and can also activate proMMP-13 [16]. MT1-MMP degrades native type I collagen, fibronectin, laminin, fibrin, gelatin and cartilage proteoglycan core protein [2] and has also been classified as an interstitial collagenase [3]. MMP-1 is the most ubiquitously expressed interstitial collagenase [3]. MMP-1 cleaves fibrillar collagens including collagens types I, II and III, resulting in cleavage products that are rapidly denatured at body temperature to gelatin, the substrate preferred by the gelatinases (MMP-2 and MMP-9) [2]. MMP-1 is produced by a wide variety of normal cells (eg stromal fibroblasts, macrophages and endothelial cells) [3], and is involved in tissue remodelling and repair [3,17]. It has been implicated in matrix invasion by the MDA-MB-231 HBC cells *in vitro *[18,19], but has a low incidence of expression in the tumour cells of breast carcinomas [2,11]. MMP-3 is a member of the stromelysin sub-family, which show broad substrate specificity, degrading type IV collagen, laminin, fibronectin and proteoglycans. MMP-3 is expressed in areas of tissue growth [20], focally expressed around invasive cells in the stromal component of breast tumours, and expressed in both benign and malignant breast phenotypes [19].

As MT1-MMP, MMP-1 and MMP-3 have all been implicated in the processes involved in human breast cancer, this study utilised HBC cell lines both in culture and grown as xenografts in nude mice, to investigate gene expression changes of MT1-MMP, MMP-1 and MMP-3 *in vitro *and *in vivo *using *IS*-RT-PCR. In order to demonstrate *IS*-RT-PCR detection to examine MMP expression level changes, we utilised Concanavalin A (Con A), an agent known to modulate MMP expression, and to upregulate at least one MMP [15,21,22], in our *in vitro *studies. Nude mouse xenografts are heterogenous, containing neoplastic human parenchyma with mouse stroma and vasculature with many histologic features of the original tumour maintained [23]. As xenograft growth varies with tumour type, the ability to mimic human tumours *in vivo *provides a reproducible model system that can be correlated to the human disease state. We have previously applied the *IS*-RT-PCR technique to examine *in vitro *gene expression of MT1-MMP in MDA-MB-231 HBC cells [21], while others have examined expression of MMP-2, MMP-9 and their inhibitors TIMP-1 and TIMP-2 in cervical cancer [24]. Thus the objective of this study was to utilise the unique *IS*-RT-PCR methodology to examine the localised gene expression of members of the MMP family of proteases implicated in breast cancer. Our findings here with *IS*-RT-PCR demonstrate the detection of *in vitro *and *in vivo *gene expression patterns of MMPs in HBC cell lines, and suggest a contribution from the stroma to MMP expression in breast carcinomas.

## Methods

### Cell culture

HBC cell lines MDA-MB-231, MCF-7, ZR-75-1 and MDA-MB-453, were originally obtained from the ATCC (Virginia, USA) and maintained by the Lombardi Cancer Center Shared Cell Culture Resource. They were grown as a monolayer culture in RPMI 1640 media (Invitrogen, USA) supplemented with 10% foetal calf serum (Biowhittaker, USA) in the presence of 5% CO_2_. Cells (approx. 1 × 10^5 ^cells/slide) were passaged onto two eight-chambered chamber slides (Lab-Tek, Nunc, USA) and allowed to attach for 8–12 hrs. Cells were then grown for a further 16–24 hrs in RPMI 1640 with 10% foetal calf serum in the presence or absence of Con A (25 μg/ml, Sigma, USA).

### Xenograft tissue

Tumour xenografts of the MDA-MB-231, MDA-MB-435, MCF-7 and Hs578T HBC cell lines were xenografted into intact female mice as described previously [25]. Animal studies were conducted in accordance with the National Institutes of Health Guide for the Care and Use of Laboratory Animals with approval of the Georgetown University Medical Center Animal Ethics Committee. The tissue was excised, and material from the xenografted tumours fixed in 10% neutral buffered formalin (Fisher Scientific, USA) and embedded in paraffin for routine histological analysis. The Lombardi Cancer Center Tissue Resource performed sectioning of the paraffin blocks, and tissue sections of 5 μm were mounted onto Pro-Bond +/- slides (Fisher Scientific, USA).

### Pre-treatment of sections

Following sectioning, slides were placed under vacuum overnight with desiccant. Our previously described RNA ISH procedure [26] was modified as follows. All slides were de-waxed in xylene (4 × 10 min), washed in ethanol (100%, 3 × 3 mins), and rehydrated through a graded ethanol series (85%, 70% and 50% in 0.9% NaCl × 2 min each). Sections were then washed in saline (0.9% NaCl, 1X × 2 min) and PBS (1X × 2 min) prior to fixation with 4% paraformaldehyde (PFA; 250 ml H_2_O, 1 g NaOH, 10 g PFA, 1.7 g sodium acetate pH 6.5) for 20 minutes. Following fixation, slides were rinsed in 2X PBS (2 mins), followed by Proteinase K digestion (10 mg/ml; Roche Diagnostics, USA; 25 ml 1 M Tris pH 8.0, 250 μl 10 mg/ml Proteinase K, 25 ml 0.5 M EDTA pH 8.0, 200 ml H_2_O). Slides were then rinsed in 2% glycine (2 mins) and re-fixed in 4% PFA (10 mins) to ensure complete deactivation of Proteinase K. Slides were rinsed in triethanolamine (TEA, Sigma, USA; 0.1 M pH 8.0 × 3 min) in preparation for further digests.

### RNase digestion

Following pre-treatment all slides were rinsed in RNase buffer (10 mM HEPES, 20 mM NaCl, 1 mM EDTA) without enzyme (2 × 2 mins). Negative control sections undergoing RNase digestion were then overlayed with RNase Digest mixture containing RNase cocktail (8 mg/ml RNase A and 160 U/ml RNase T1 final volume, Bresatec, Australia) in RNase buffer, placed in a humid chamber and incubated at 37°C for 8 hrs. Untreated slides were overlayed in RNase buffer and also incubated at 37°C for 8 hrs.

### DNase pre-treatment

Prior to DNase digest, all slides were rinsed in DNase buffer (0.1 M C_2_H_3_O_2_Na, 5 mM MgSO_4 _pH 5.0, 2 × 2 min). DNase buffer (200 μl) containing 1.5 U/ml final volume of RNase-free DNase (Roche Diagnostics, USA) was added to each slide and these were incubated overnight at 37°C in a humid chamber. After incubation the slides were rinsed thoroughly in DNase buffer without enzyme, followed by washes in sterile water and dehydration through a graded ethanol series (50%, 70%, and 90% in 0.9% saline, 100% × 3 mins each).

### Primers

Primers encoding MT1-MMP, MMP-1 and MMP-3 (Bresatec, Australia) were designed using the Amplimer program, product design checked by the Amplify v1.2 program, and BLASTed (NCBI, USA) for homology to the mRNA of these genes, with significant sequence homology demonstrated between the human and mouse MMPs examined. Primers encoding human β-actin were used as a positive control (Research Genetics, USA). Primer sequences are listed in Table 1. All primers were tested using traditional RT-PCR on mRNA extracted from MDA-MB-231 and MCF-7 HBC cells using the High Pure RNA Isolation Kit (Roche Diagnostics, USA). Reverse transcription was performed using 20 ng of isolated mRNA, by AMV reverse transcriptase (Promega Corporation, USA) at 50°C for 30 minutes, followed by PCR amplification using Taq DNA Polymerase (Perkin-Elmer Corporation, Applied Biosystems Division, USA). The linear phase of amplification was determined to be between cycles 20–25 using a standard two-step PCR cycle.

### In situ-Reverse Transcription-Polymerase Chain Reaction (IS-RT-PCR)

Prior to the *IS*-RT-PCR procedure Gene-Frame gaskets (Advanced Biotechnologies, UK) were placed around the sections. *IS*-RT-PCR was performed on consecutive sections for each xenograft: section A) RNase/DNase negative control; B) β-actin positive control; C) MT1-MMP assay slide; D) MMP-1 assay slide; and E) MMP-3 assay slide. Reverse transcription was performed as previously reported [18] with the following modifications: dCTP concentration was 100 μM, biotin-dCTP 80 μM, MnCl_2 _10 mM and 7.5 U of rTth DNA Polymerase (Perkin-Elmer Corporation, Applied Biosystems Division, USA) was used in the 30 μl mix added to each slide. Linear amplification efficiency was determined using traditional RT-PCR as outlined above, to be between cycles 20 and 25. For the amplification step the initial denaturation was at 95°C for 4 minutes, the MgCl_2 _concentration was 15 mM, and 30 μl were added to each slide. Amplification was performed for 20 cycles.

### Detection

Post *IS*-RT-PCR detection was performed as reported previously [21] with the following modifications: Incubation in the NBT/BCIP/ASB solution was for 15 minutes, slides were counterstained with eosin (Australian Biostain Pty Ltd, Australia) and mounted in Ultramount (Fronine, Australia). Consistency of observed gene expression in consecutive tumour sections demonstrated the reliability of the *IS*-RT-PCR technique and as expected, all cells were found to express the ubiquitous β-actin gene.

### Signal quantitation

**Figure 1:**
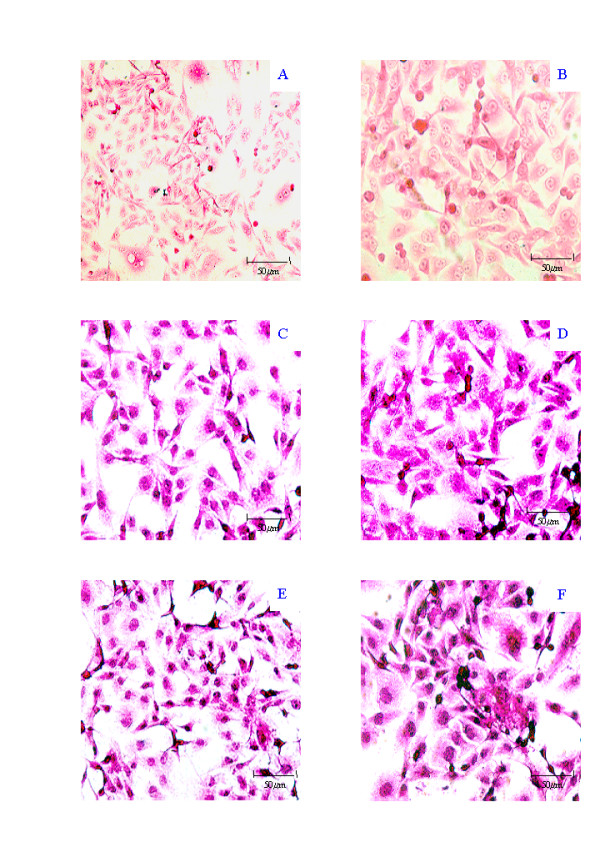
Representative photomicrographs of *IS*-RT-PCR analysis of Con A effects in MDA-MB-231 cells counterstained with Eosin. Negative controls include, RNase/DNase treated sections (A) and No RT (B). β-actin staining provided a positive Control without (C) or with (D) Con A treatment. MT1-MMP levels before (E) and after (F) Con A treatment are also shown. Photographs were on an Nikon Eclipse TE300 Microscope with a Nikon F-600 camera attachment and are × 40 (except for A, which is × 20).

**Figure 2:**
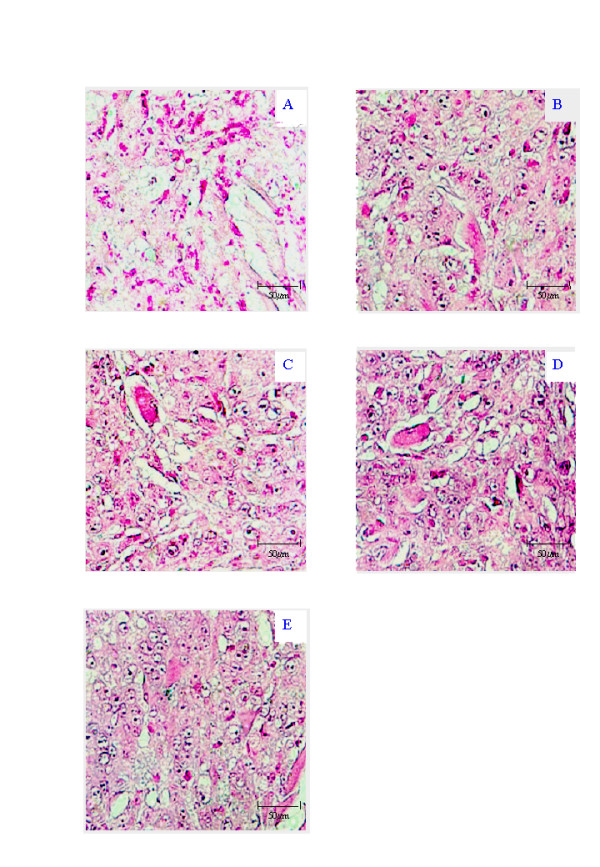
Representative photomicrographs of *IS*-RT-PCR results of MCF-7 xenograft showing the RNase/DNase negative control (A), positive control β-actin (B), MT1-MMP gene expression (C), MMP-1 gene expression (D) and MMP-3 gene expression (E). All photographs ×40 taken on an Olympus Microscope with a Nikon F-600 camera attachment.

**Figure 3:**
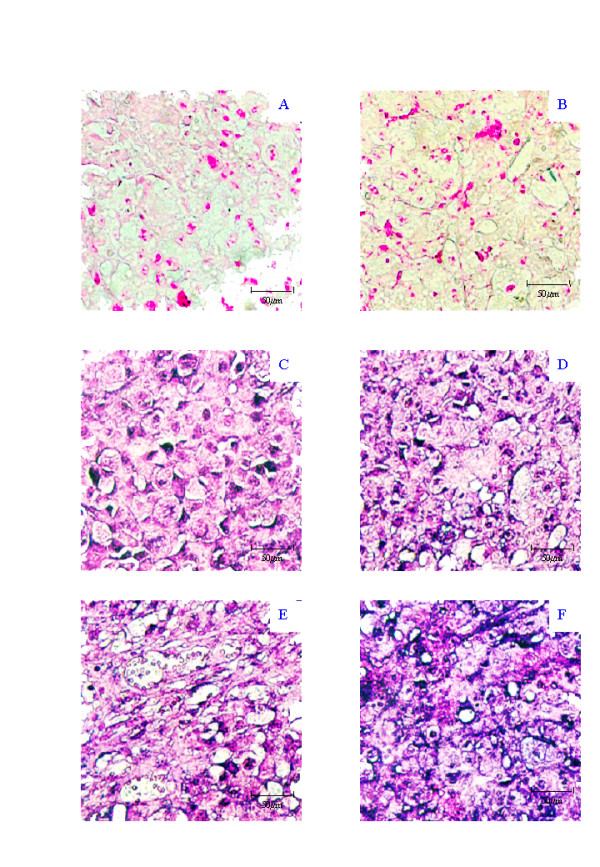
Representative photomicrographs of *IS*-RT-PCR results of MDA-MB-435 xenograft showing the No RT control (A), RNase/DNase negative control (B), positive control β-actin (C), MT1-MMP gene expression (D), MMP-1 gene expression (E) and MMP-3 gene expression (F). All photographs ×40 taken on an Olympus Microscope with a Nikon F-600 camera attachment.

The level of signal intensity was assigned a value from zero to four (0+ to 4+). Slides showing no purple precipitation and only eosin counterstain were assigned a value of zero (0+), whilst cells stained with precipitate were assigned a value between one and four (1+ to 4+). The observed signal intensity was assessed based on the observed level of signal intensity and tissue morphology using light microscopy. Visual characteristics including the overall level of precipitation, direct comparison of the entire tissue area to compensate for background, and the number and intensity of cells exhibiting purple precipitation were also considered. In addition, a coded labelling system was used to ensure that slide examination was unbiased. *In vitro *signal intensity following exposure to Con A and cytokines was compared to the untreated or basal level allowing an estimate of up/down-regulation to be determined. The *in vivo *xenograft material was histologically assessed and scored from 0+ to 4+ (with 0 = no expression; 1+ = low expression; 2+ = moderate expression; and 3+ = strong expression). We compared the relative levels of gene expression of each MMP within the xenografts analysed to controls. Following independent examination of slides and sections by three individuals the signal level intensity was averaged. The photographs presented in Figures 1, 2 and 3 are of the same area within consecutive sections and are therefore a representation of the overall observed gene expression levels.

## Results

### Primer specificity

Primers were tested using traditional RT-PCR on mRNA extracted from cultured MDA-MB-231 cells. Bands of the appropriate size for β-actin (289 bp), MT1-MMP (274 bp), MMP-1 (330 bp) and the MMP-3 (330 bp) transcripts were visualised by ethidium bromide agarose gel electrophoresis (not shown). For *IS*-RT-PCR two negative controls were used to ensure assay validity. The "No RT" control was used as a measure of the detection system specificity. Lack of precipitation in this control ensures that a positive signal is due to expression of the gene of interest. The RNase/DNase control was used for two reasons, to control for false positive results due to insufficient genomic DNA digestion, and to ensure that there was no signal due to any residual biotin-dCTP in the tissue section. Examples of these controls are given for the MDA-MB-231 cell line (Figure 1A and 1B), where the only colouration in the slides is due to the eosin counterstain, and purple precipitation which would be indicative of positive gene expression signal, is not observed. Primers amplifying β-actin mRNA were used as a positive control for demonstration of mRNA preservation in the tissue, and the ability to detect gene expression. We found a clear positive signal, the purple precipitate localised to all cells examined, demonstrating β-actin expression. An example of this is given in Figure 1C for the MDA-MB-231 cell line.

### Induction of in vitro MMP gene expression

#### IS-RT-PCR results following Con A treatment

Expression levels of the three MMPs were examined with respect to β-actin in the MDA-MB-231, and MCF-7 cell lines, with or without exposure to Con A. In the MDA-MB-231 cell line, MT1-MMP expression was increased from 2+ to 4+ by Con A treatment, while MMP-1 and MMP-3 remained unchanged at 2+ following exposure to Con A. MT1-MMP induction by Con A in the MDA-MB-231 cell line is shown in Figure 1E and 1F. In the MCF-7 cell line, the MMP-1, MMP-3 and β-actin expression levels also remained unaffected by Con A at 2+, however, the MT1-MMP expression was decreased by Con A from 3+ in the absence of Con A to 1+ in the presence of Con A (not shown).

#### Adaptation of IS-RT-PCR to paraffin tissue

We adapted the *IS*-RT-PCR protocol for analyses of these MMPs in paraffin embedded xenografts of MCF-7, MDA-MB-231, MDA-MB-435, and Hs578T HBC cell lines grown subcutaneously in the mammary region of nude mice. Primary xenograft material from each cell line (except MDA-MB-231) were examined, as were metastatic deposits of MDA-MB-231 cells in the spleen and mesentery. The primary MDA-MB-231 tumour was unavailable for examination. A summary of the results obtained from the xenograft tissue is given in Table 2.

In the MCF-7 xenograft, low expression of MT1-MMP, MMP-1 and MMP-3 was observed in the tumour cells (1+) as indicated by the faint purple precipitation (Figure 2C, 2D, 2E). A low level of gene expression for MT1-MMP, MMP-1 and MMP-3 (1+) was also seen in the stromal compartment surrounding the MCF-7 cells (Figure 2C, 2D, 2E). However, β-actin expression was low in the stromal component (1+) and at a moderate level in the tumour component (2+) suggesting that the relative stromal levels are even higher (Figure 2B). A low level of expression of β-actin was consistent between the two compartments (1+). The MDA-MB-435 No RT control (Figure 3B) served as the No RT control section for the MCF-7 experiment, with the experiments run concurrently. In the MDA-MB-435 cell line xenograft, we observed moderate expression of MT1-MMP and MMP-1 (2+) (Figure 3C and 3D), and high levels of MMP-3 (3+) (Figure 3E) in the tumour cells. No expression of MT1-MMP (0+), MMP-1 (0+) or MMP-3 (0+) was observed in the stromal tissue surrounding the MDA-MB-435 cells (Figure 3D, 3E and 3F). β-actin expression was consistently moderate in both compartments (2+) (Figure 3C).

The Hs578T xenograft showed low expression of MT1-MMP (1+), MMP-1 (1+) and MMP-3 (1+) in the tumour cells. In the surrounding stromal cells, we observed low MT1-MMP expression (1+), moderate MMP-3 expression (2+) and no MMP-1 expression (0+). β-actin expression levels were consistently low in both compartments (1+) (Table 2). Although the primary MDA-MB-231 xenograft was unavailable for examination, mesenteric deposits in the spleen and mesentery of the same mouse were examined (Table 2). In the MDA-MB-231 spleen metastasis, the tumour cell showed high MT1-MMP (3+), and low MMP-3 expression (1+), but no detectable MMP-1 gene expression (0+). No detectable expression of MT1-MMP, MMP-1 and MMP-3 was observed in the host stromal cells (0+). β-actin gene expression was consistent between the two compartments at a moderate level (2+) (Table 2). In the mesenteric metastasis, the MDA-MB-231 tumour cells showed a moderate expression of both MT1-MMP and MMP-1 (2+), and low expression of MMP-3 (1+). In contrast to the splenic metastasis, the host stromal tissue surrounding the mesenteric metastasis showed low expression of MT1-MMP (1+), MMP-3 (1+) and moderate MMP-1 expression (2+). Again, β-actin was consistent in both compartments at a moderate level (2+) (Table 2).

## Discussion

The central aim of investigations into molecular carcinogenesis is the identification of gene products involved in cancer progression during which tumour cells acquire the capacity for invasion with resulting metastasis [27]. Metastasis is an active process involving the altered attachment of the tumour cell to the basement membrane, localised degradation of connective tissue, and migration through stromal tissue [28,29]. Such matrix degradation is mediated by members of several proteinase families (the serine, cysteine, aspartate proteinases and MMPs), with substantial tissue destruction being carried out by members of the MMP family [3]. The expression of members of the MMP family and their inhibitors, the TIMPs, have been examined in both physiological and pathological conditions by many methods both *in vitro *and *in vivo*, including the use of total RNA from tissues and cell lines, Northern analysis, RT-PCR *in situ *and standard *in situ *hybridisation (ISH) [13,21,24,30-33]. Xenografts of cell lines representing increased progression of breast cancer were examined in the present study using the *IS*-RT-PCR technique. The samples ranged from the poorly invasive, estrogen-receptor positive cells (MCF-7), through to examples of more aggressive, estrogen-receptor negative human breast carcinoma (MDA-MB-231, MDA-MB-435, Hs578T) [25]. We analysed and compared the localised expression of MT1-MMP, MMP-1, MMP-3 and β-actin, in both the tumour parenchyma and the surrounding stroma. Both the MDA-MB-231 splenic and MDA-MB-435 xenografts demonstrate no detectable MMP gene expression in the stromal compartment. This highlights that although significant sequence homology exists between the human and mouse MMPs examined, minor differences may influence the detection of gene expression. Localised gene expression in both the epithelial tumour cells and the stroma compartments in the remaining xenografts however, demonstrate the detectable homology between human and mouse for the *IS*-RT-PCR primer sequences used.

MT1-MMP analysis of the HBC xenografts demonstrated mRNA in the tumour cells of all of the samples examined. *In vitro*, we have previously demonstrated MT1-MMP expression in MCF-7 cells by *IS*-RT-PCR [21], however Northern analysis of MCF-7 cells failed to find MT1-MMP in MCF-7 cells, whereas more invasive HBC cell lines showed MT1-MMP expression in concordance with their ability to be induced by Con A to activate MMP-2 [12,34,35]. MT1-MMP has also been shown to correlate with increased invasion and to enhance migration of MCF-10A epithelial cells [6,12,36], and transfection of MT1-MMP into MCF-7 cells stimulates higher migration and invasiveness [37,38], and also stimulates VEGF production, angiogenic stimulation, and xenograft take rate in nude mice [39,40]. Presumably the *IS*-RT-PCR method is more sensitive than Northern analysis, as may be expected. Indeed, we detected lower levels of MT1-MMP in the MCF-7 xenografts than in the majority of other xenografts derived from more invasive HBC cells. More recently, using isolated RNA for quantitative analyses, we were unable to detect MT1-MMP in MCF-7 derived xenografts, but consistent with our observations here, increasing levels of MT1-MMP was detected in MDA-MB-231 derived xenografts as compared to the parental cells [13]. The reasons for these differences are not clear and require further experimentation, but it is important to note that the cultured MCF-7 cells would have received estrogenic stimuli from both the phenol red and foetal calf serum in the medium [41].

MT1-MMP overexpression in the mammary gland results in abnormalities including hyperplasia, fibrosis, lymphocytic infiltration and adenocarcinoma [42], suggesting a pivotal role for MT1-MMP in carcinogenesis. However, *IS*H analyses of human breast carcinomas have primarily localised MT1-MMP mRNA expression to the stromal cells [11,29,43-45], although one study found localised MT1-MMP in the tumour [46]. An immunocytochemical analysis showed expression of MT1-MMP in both tumour and stromal cells [47]. More recently, one study by Bisson et al, combining *IS*H and IHC data has co-localised MT1-MMP to the α-smooth muscle positive myofibroblast cells in close contact with tumour cells [48]. Our studies certainly show the potential for the cell lines examined to make MT1-MMP, even the relatively well-conserved cell lines such as MCF-7. Although it is acknowledged that *in vitro *propagation of cell lines may alter some genetic pathways, it is also apparent that cell lines and tumour samples have distinctive gene expression patterns in common [49]. Being ER-positive, and predominantly epithelial, MCF-7 cells far better represent what one may find clinically in breast cancers than the other cell lines examined here [50]. The invasive HBC cell lines show a mesenchymal-like phenotype that may have resulted from epithelial-mesenchymal transition (EMT) in breast carcinoma. EMT is thought to have occurred in the MDA-MB-231, MDA-MB-435 and Hs578T cell lines [51]. The demonstration of MT1-MMP expression observed in the stromal component is not totally unexpected. MT1-MMPs ability to stimulate invasion and metastasis in *in vitro *systems along with the estrogen receptor status of the cells is well documented as described above. Also, the basal levels observed between the MCF-7 and more invasive HBC cell lines may also be of impact in respect to the higher invasive potential of the cells.

MMP-1 mRNA was detected in the tumour cells of five of the six xenografts examined, the exception, surprisingly, being the MDA-MB-231 splenic metastasis. This is unexpected since MDA-MB-231 cell overexpress MMP-1 in culture [52], and these cells have a mutation/polymorphism in the promotor region which drives strong MMP-1 expression [18]. This transcriptional repression has not been found to occur extensively in breast cancer [18]. The reasons for suppression of the MMP-1 expression in splenic metastasis are not clear, but could include a strong repression, possibly due to altered signalling from the stroma. The splenic metastasis stroma was notably also lacking all three MMPs examined, but showed a strong β-actin signal in both compartments. One cannot rule out the possibility of selective aberrant loss of MMP mRNA rather than the β-actin, however, this is unprecedented. Further analysis of splenic metastases of the MDA-MB-231 cells, and primary human tumours, would be warranted. MMP-1 has been previously demonstrated to be equally expressed *in vitro *in both MCF-7 and MDA-MB-231 cells [53], although in a similar study using Northern analysis we found selective expression in MDA-MB-231 cells but not MCF-7 [51]. Increased production and secretion of MMP-1 has been correlated with increased metastatic potential [52,54]. More recently, Bachmeir *et al *have demonstrated a cell density-dependent regulation of gene expression of several MMPs in HBC cell lines [31]. Increased cell density resulted in decreasing levels of both MMP-1 and MMP-3 expression levels, with a resultant decreased invasion concurrent with invasive potential [31-33]. Increased expression of MMP-1 has been reported on contact with Matrigel [55], and also in response to fibronectin fragments [56], attesting to the potential regulation of this MMP by the microenvironment. *In vivo*, MMP-1 expression has been demonstrated in the stroma of nine of thirty-four breast carcinomas [11] when examined by *IS*H, localised to stromal constituents during tumour formation [57], and to be upregulated in the stromal component of ductal carcinomas when examined by *IS*H and IHC [57-59]. Thus, again, our observation that MMP-1 expression was absent in the stroma of three out of six HBC xenografts appears to contrast the clinical situation. This may reflect the increased sensitivity of *IS*-RT-PCR and may also reflect the stage in tumour formation of the MCF-7 and MDA-MB-231 mesenteric xenografts that we examined.

MMP-3 mRNA was localised to the tumour component in all six xenograft samples examined. *In vitro*, MMP-3 mRNA has been demonstrated in the MDA-MB-231 HBC cell line [55], where it is stimulated by fibroblasts via the extracellular matrix metalloproteinase inducer (EMMPRIN) [60], and can enhance tumourigenicity and migration [54]. *In vivo*, MMP-3 is centrally involved in mammary gland development [61], and has been demonstrated to promote tumour initiation and formation in the tetracycline-regulated mouse mammary model [62,63]. IHC and *IS*H *in vivo *analysis has demonstrated MMP-3 in both the tumour and stroma cellular compartments of both invasive and non-invasive tumours, with the level of stromal expression increasing with tumourigenicity [59,64,65], and in the extracellular matrix adjacent to breast tumours [66]. Our observation of MMP-3 gene expression in the stroma of four out of six of the xenografts examined, and in particular in the Hs578T xenograft, further support a role for this MMP in breast cancer.

Although HBC cell lines have been demonstrated to express certain MMPs, this is less evident *in vivo *where, as detailed above, the surrounding stromal cells contribute to much of the MMP activity. Important differences in MMP expression between the stromal cells recruited by each cell line and the tumour cells themselves, were detected in the current study. The MCF-7 xenograft stroma demonstrated low stromal MT1-MMP, MMP-1 and MMP-3 expression. MT1-MMP expression was observed at a lower level in the stroma associated with the MDA-MB-231 mesenteric metastasis. In the Hs578T xenograft stroma, MMP-1 was absent while MMP-3 was observed at a higher level in the stroma as compared to the tumour cells. No stromal expression of the three MMPs examined was observed in either the MDA-MB-231 spleen metastasis or the MDA-MB-435 xenografts. The level of stromal gene expression will depend on signals from the different HBC lines in the primary site, and also on the different responsivities in the different host sites for the splenic and mesenteric metastases. In regard to the primary site, overall recruitment of the stroma will differ among the HBC lines; however, β-actin mRNA detection was used to normalise the data. We did detect lower levels of β-actin in the Hs578T xenograft, and indeed higher levels of β-actin in the tumour cell component of the MCF-7 xenograft. However, this does not appear to have influenced the detection of the MMPs as indicated by the moderate levels of all MMPs examined in both compartments in the MCF-7 xenograft, and the moderate level of MMP-3 in the Hs578T tumour compartment.

## Conclusion

These data combined indicate communication, and in particular HBC cell-line specific communication, between the epithelial and stromal cellular compartments for MMP production and utilisation. Considerable work has been performed *in vitro *to examine the effects of different HBC cell lines on MMP expression by co-culture with fibroblasts and other factors known to be produced by the tumour cells which mediate induction of MMPs (eg Con A, TPA, EMMPRIN) [12,15,21,31-33,53,60]. These and other studies, implicate increasing MMP levels associated with both epithelial tumour cells and surrounding stromal cells, in association with increasing tumourigenicity and metastatic potential [12,15,21,24,53,54,60,67]. Our *in vivo *data indicate that, the MDA-MB-231, MDA-MB-435 and Hs578T HBC cell lines are able to influence MMP gene expression in the surrounding stroma. This is reinforced by a recent study demonstrating the influence of tumour associated fibroblasts on MCF-7 tumour cells to activate MMP-2 *in vitro *and increase tumour size *in vivo *[68]. These data combined indicate *IS*-RT-PCR may serve as a more sensitive, one-step procedure for the examination of MMP gene expression in human breast cancers.

## Competing interests

The author(s) declare that they have no competing interests.

## Authors' contributions

L.M.H. performed all *in vitro *and *in vivo *studies and drafted the manuscript. E.W.T. contributed toward the design of the study and manuscript finalisation. A.E.O.T. contributed toward the design of the *in situ *sample preparation. R.E.I. contributed toward data management and manuscript finalisation. M.G.I. participated in the conception and design of the study. L.R.G. participated in the conception and design of the study and its coordination. All authors read and approved the final manuscript.

## Pre-publication history

The pre-publication history for this paper can be accessed here:



## Figures and Tables

**Table 1:** Primer sequences encoding the human β-actin, MT1-MMP, MMP-1 and MMP-3 genes.

**Gene**	**Primer Sequence**
β-actin sense	5'- ACC CAC ACT GTG CCC ATC TA
β-actin antisense	5'- CGG AAC CGC TCA TTG CC
MT1-MMP sense	5'- AGT GGA TGG ACA CGG AGA AT
MT1-MMP antisense	5'- TCC ATC CAT CAC TTG GTT AT
MMP-1 sense	5'- AGC GTG TGA CAG TAA GCT AA
MMP-1 antisense	5'- GTT TTC CTC AGA AAG AGC AGC AT
MMP-3 sense	5'- GTC TCA AGA TGA TAT AAA TG
MMP-3 antisense	5'- AAT TGA TTT CCT TTA AAA ATG A

**Table 2:** Summary of *IS*-RT-PCR results obtained for expression of β-actin, MT1-MMP, MMP-1 and MMP-3 in HBC cell line derived xenograft tissues. The intensity is a value from zero to four (0+ to 4+) based on the overall level of signal intensity observed throughout the entire section.

Cell Line	β-actin	MT1-MMP	MMP-1	MMP-3
MCF-7	Tumour 2+	Tumour 1+	Tumour 1+	Tumour 1+
	Stroma 1+	Stroma 1+	Stroma 1+	Stroma 1+
MDA-MB-231	Tumour 2+	Tumour 3+	Tumour 0+	Tumour 1+
Spleen	Stroma 2+	Stroma 0+	Stroma 0+	Stroma 0+
MDA-MB-231	Tumour 2+	Tumour 2+	Tumour 2+	Tumour 1+
Mesenteric	Stroma 2+	Stroma 1+	Stroma 2+	Stroma 1+
MDA-MB-435	Tumour 2+	Tumour 2+	Tumour 2+	Tumour 3+
	Stroma 2+	Stroma 0+	Stroma 0+	Stroma 0+
Hs578T	Tumour 1+	Tumour 1+	Tumour 1+	Tumour 1+
	Stroma 1+	Stroma 1+	Stroma 0+	Stroma 2+
